# SMARCA4-Deficient Carcinomas of the Small Intestine: A Systematic Review

**DOI:** 10.3390/curroncol33020107

**Published:** 2026-02-10

**Authors:** Aaqid Syed, Yanis Boumber, Midhun Malla

**Affiliations:** 1 Mobile Infirmary Medical Center, Mobile, AL 36607, USA; aaqid.syed@infirmaryhealth.org; 2 Thoracic Medical Oncology Section, Phase 1 Unit, O’Neal Comprehensive Cancer Center, UAB Heersink School of Medicine, Birmingham, AL 35233, USA; yboumber@uabmc.edu; 3 Heersink School of Medicine, University of Alabama at Birmingham, Birmingham, AL 35233, USA; 4 GI Medical Oncology, O’Neal Comprehensive Cancer Center, University of Alabama at Birmingham, Birmingham, AL 35233, USA

**Keywords:** SMARCA4-deficient carcinoma, rhabdoid features, SWI/SNF complex, small intestine

## Abstract

**Purpose**: SMARCA4-deficient carcinomas are rare, aggressive malignancies characterized by loss of BRG1, a core component of the SWI/SNF chromatin remodeling complex. These typically arise in the chest, but recent case reports suggest rare involvement of the gastrointestinal tract, particularly the small intestine. This review aims to consolidate the available literature on SMARCA4-deficient carcinomas of the small intestine, highlighting their clinical, histopathological, molecular, and therapeutic features. **Design**: A systematic review of PubMed was conducted through March 2025 to identify all published cases of primary SMARCA4-deficient carcinomas of the small intestine. Inclusion criteria required immunohistochemical or molecular confirmation of SMARCA4/BRG1 loss. Studies describing metastases, non-small intestine primaries, or lacking molecular data were excluded. Ten eligible cases were analyzed in detail. **Results**: Patients were predominantly male (9 out of 10) with a median age of 54 years. Most carcinomas arose in the duodenum and patients frequently presented with nonspecific symptoms and advanced-stage disease. Histologically, tumors demonstrated undifferentiated or rhabdoid features with high mitotic activity and extensive necrosis. Immunohistochemistry confirmed loss of SMARCA4, while several cases also showed SMARCA2 loss. Molecular profiling revealed congruent mutations in TP53 and CTNNB1, among oncogenic drivers. While some patients with localized disease achieved prolonged survival after surgery and adjuvant chemotherapy, those with metastatic disease had limited responses to immune checkpoint inhibitors, despite PD-L1 positivity in a subset. Overall survival ranged from 2 to 29 months. **Conclusions**: SMARCA4-deficient carcinomas of the small intestine represent a distinct, high-grade malignancy with poor prognosis and limited therapeutic options. Prompt recognition, SMARCA4 testing, and referral to specialized centers are essential. Prospective studies are needed to guide therapy and explore targeted approaches in this challenging carcinoma subtype.

## Simple Summary

Cancers of the small intestine are rare, and some aggressive forms are difficult to diagnose and treat. One such form involves loss of a gene called SMAR-CA4, which normally helps control how cells grow and divide. When this gene is lost, cancers tend to grow quickly and respond poorly to standard treatments. Most knowledge about SMARCA4-deficient cancers comes from tumors in the chest and very little is known about those arising in the small intestine. In this study, we reviewed all published cases of SMARCA4-deficient small intestine cancer to better understand how these tumors present, how they look under the microscope, how they behave and how patients respond to treatment. We found that most patients were middle-aged men who presented with advanced disease and vague symptoms such as abdominal pain or bowel obstruction. These cancers showed highly aggressive features and were associated with short survival in most cases. Surgery combined with chemotherapy offered longer survival only in patients diagnosed early. Recognizing this rare cancer early and testing for SMARCA4 loss is critical, as it may guide treatment decisions and referral to specialized centers.

## 1. Introduction

The SMARCA4 [SWItch/Sucrose Non-Fermentable complex (SW1/SNF)-related, matrix-associated, actin-dependent regulator of chromatin, subfamily A, member 4] gene encodes BRG1, a core catalytic subunit of the SWI/SNF chromatin remodeling complex, which regulates gene transcription by modifying chromatin remodeling [1,2]. SMARCA4 is a carcinoma suppressor that plays a critical role in cellular differentiation and genomic stability [3]. Loss of expression of SWI/SNF complex proteins, either through genetic mutations or epigenetic mechanisms have been implicated in the pathogenesis of a wide range of carcinomas [4]. SMARCA4 has been shown to regulate developmental processes, transcriptional regulation, DNA repair, cell cycle, and carcinoma [5]. This has been reported across multiple carcinoma types, including lung, thoracic, and gynecologic malignancies [6,7,8,9]. It was initially identified in pediatric malignant rhabdoid carcinomas and later recognized as a defining molecular event in small cell carcinoma of the ovary (hypercalcemic type), as well as a dedifferentiation mechanism in a subset of endometrioid carcinomas of the uterus [10,11].

While most SMARCA4-deficient carcinomas arise in the thoracic cavity, rare presentations involving the gastrointestinal (GI) tract have been increasingly reported, particularly in the form of undifferentiated or rhabdoid carcinomas [12,13]. Undifferentiated carcinoma of the small intestine is an exceedingly rare malignancy characterized by sheets of anaplastic cells lacking definitive glandular, squamous, neuroendocrine, or sarcomatoid differentiation [14]. When these carcinomas exhibit loss of SMARCA4, they are categorized as SMARCA4-deficient undifferentiated carcinomas (SMARCA4-dUTs) which poses significant therapeutic challenges [12]. They are often managed with systemic chemotherapy regimens, typically based on platinum-based therapies such as FOLFOX (folinic acid, fluorouracil, and oxaliplatin). Despite these interventions, median overall survival remains limited, underscoring the need for more effective treatment strategies [13].

Given their rarity, there is no consensus on diagnostic criteria or approved effective treatment, and the prognostic implications remain unclear. Interestingly, recent pre-clinical studies suggest that targeting SMARCA2 (SMARCA4 paralogue) is highly efficacious in SMARCA4-mutant carcinoma due to synthetic lethality effects, representing a novel therapeutic approach [15]. As such, it is crucial to compile and analyze the existing literature to better understand the behavior of SMARCA4-deficient carcinomas of the small intestine. This review aims to summarize the reported cases in the literature, highlight common patterns, and contribute to a growing body of knowledge regarding these aggressive neoplasms.

## 2. Methodology

A comprehensive literature review was conducted using the PubMed and Google Scholar database to identify all reported cases of SMARCA4-deficient carcinomas involving the GI tract, with a particular focus on the small intestine. The search was performed using the following keywords and Boolean combinations: (“SMARCA4” OR “BRG1”) AND (“small intestine carcinoma” OR “small bowel carcinoma” OR “gastrointestinal undifferentiated carcinoma” OR “SMARCA4-deficient carcinoma” OR “rhabdoid carcinoma”). All available articles published from database inception through March 2025 were screened.

**Figure 1:**
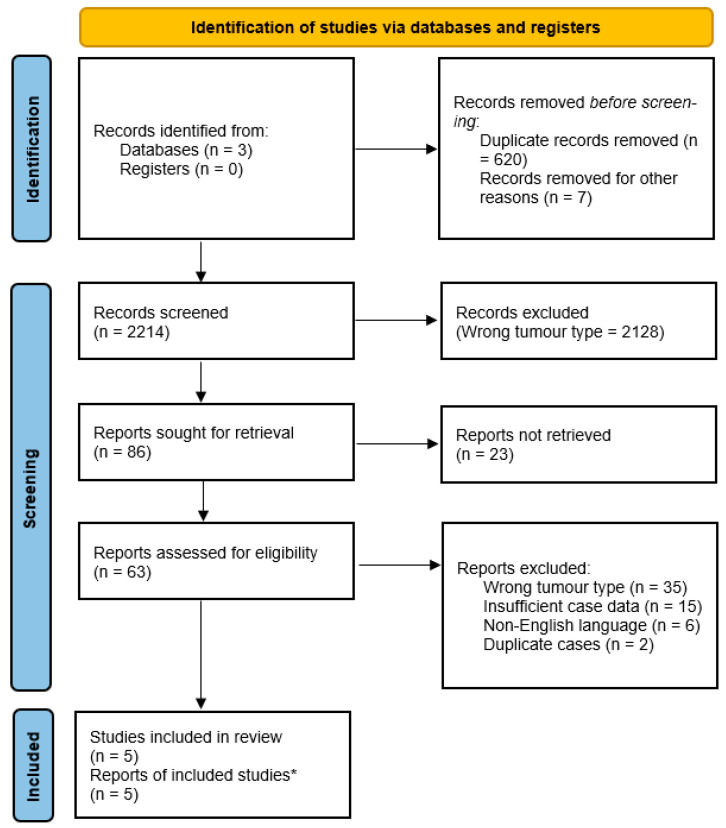
PRISMA 2020 flow diagram of study selection. * Several reports described more than one eligible patient, resulting in a total of ten individual cases analyzed.

This systematic review was conducted in accordance with the Preferred Reporting Items for Systematic Reviews and Meta-Analyses (PRISMA) guidelines. A PRISMA flow diagram summarizing the study selection process is provided in Figure 1.

Studies were eligible for inclusion based on the following:

• Reported on primary GI carcinomas, specifically involving the small intestine (duodenum, jejunum, or ileum);
• Documented loss of SMARCA4/BRG1 protein expression confirmed via immunohistochemistry or molecular analysis.

Articles were excluded based on the following:

• Described non-GI tract carcinomas (e.g., lung, ovarian, or soft tissue SMARCA4-deficient malignancies);
• Involved small intestine metastases from other primaries;
• Focused on gastric, colonic, or rectal carcinomas without small intestine involvement;
• SMARCA4 status was not confirmed or was reported as intact/wild-type.

The outcomes for which data were sought included clinical presentation, diagnostic pathways and delays, treatment strategies, and reported patient outcomes such as survival status, disease progression, recurrence, and duration of follow-up when available. Additional variables extracted included patient demographics (age and sex), tumor location within the small intestine (duodenum, jejunum, or ileum), histopathologic characteristics, immunohistochemical and/or molecular findings confirming loss of SMARCA4 (BRG1) expression, study type (case report or case series), and year of publication. When information was missing or unclear, data were recorded as not reported and no assumptions were made.

No formal risk of bias or methodological quality assessment was performed. This decision was based on the extreme rarity of SMARCA4-deficient carcinomas of the small intestine and the exclusive inclusion of case reports and small case series, for which conventional risk-of-bias tools are of limited applicability.

Titles and abstracts were initially screened to exclude irrelevant studies, followed by full-text review to confirm eligibility. Reference lists of included articles were also manually reviewed to identify any additional relevant cases not captured in the initial search. Study selection and data extraction were independently performed by two authors. Any uncertainties during screening or data interpretation were resolved through consultation with the corresponding author. Several included publications reported more than one eligible patient. In such instances, each individual case was extracted and analyzed separately, resulting in a case-level rather than study-level analysis. This systematic review was registered in the Research Registry. The Unique Identification Number (UIN) is researchregistry2074. A separate review protocol was not prepared for this study.

## 3. Summary of Reported Cases

### 3.1. Demographics and Clinical Presentation

A total of ten patients with SMARCA4-deficient small intestine carcinoma were identified across published reports and institutional case series [12,13,16,17,18]. Clinical, histopathologic, and treatment characteristics are summarized in Table 1. The median age at diagnosis was 54 years (range: 34–76), with all cases reported in male patients except one.

Clinically, majority of the patients presented with nonspecific abdominal symptoms such as intermittent pain, bowel obstruction, or intussusception. In multiple cases (cases 3, 4, 7, 9), the disease was already metastatic at diagnosis, involving regional lymph nodes, liver, brain, or lungs. Tumor staging at diagnosis ranged widely, but stage IV was most frequently encountered, highlighting the aggressive biology and tendency for these carcinomas to present late. Smoking was the only identifiable risk factor in two patients. There was no reported association with inflammatory bowel disease, hereditary syndromes, or prior radiation therapy.

### 3.2. Tumor Location and Macroscopic Features

Anatomically, SMARCA4-deficient carcinomas most frequently arose from the duodenum (cases 3, 4, 6, 7, 8, 9) including the ampulla of Vater. There was one case involving the jejunum and three diffusely affecting the small intestine (cases 1, 2, 10). Two cases demonstrated multiorgan involvement at presentation, including the adrenal glands (case 1) and liver (case 3). Gross findings often demonstrated large heterogeneous masses with exophytic or infiltrative growth patterns, consistent with aggressive biology. Imaging details were inconsistently reported but notable findings included heterogeneous masses in both small intestine and adrenal glands (case 1), and small bowel intussusception (case 2).

### 3.3. Integrative Pathological and Molecular Profile

Morphologically, most carcinomas consisted of diffuse sheets or nests of epithelioid cells with marked pleomorphism, high mitotic activity, and extensive necrosis (Table 1). Rhabdoid morphology was a recurrent feature, often accompanied by multinucleated giant cells and occasional pseudoglandular patterns (cases 5, 6, 8, 9). All cases exhibited either undifferentiated or poorly differentiated carcinoma grade and some carcinomas were almost entirely undifferentiated (Cases 3, 4, 6).

Immunohistochemistry (IHC) revealed a consistent loss of SMARCA4 (BRG1) expression across all cases, confirming their molecular classification. Three carcinomas also demonstrated concurrent loss of SMARCA2 (cases 6–8), supporting a dual SWI/SNF complex deficiency in a subset of cases. Integrator Complex Subunit 1 (INI1) expression was retained in all evaluable cases, ruling out SMARCB1-deficient rhabdoid carcinomas. Vimentin was frequently positive, especially in those with rhabdoid morphology, suggesting epithelial–mesenchymal transition (EMT)-like features. Pan-cytokeratin expression varied considerably, while some carcinomas retained strong epithelial markers; others showed partial or complete loss, especially in undifferentiated areas. Additional IHC findings included positivity for markers such as ARID1A and mutant p53, with variable expression of EMA, CK7, CK20, and S100.

Molecular profiling, when available, revealed additional alterations including TP53, CTNNB1, ERBB3, NCOR1, MYH9, and RAD52. Tumor mutational burden (TMB) was elevated (10.56) in case 4. Mismatch repair (MMR) protein expression was retained in cases where it was tested, indicating microsatellite stability (MSS). Notably, PD-L1 expression (reported as CPS scores) was >1 in the two cases tested, with combined positive scores (CPS) ranging from 5 to 6.

### 3.4. Treatment and Response

Therapeutic approaches varied depending on the disease stage (Table 1). Of the ten cases, three patients (cases 2, 5, and 6) were diagnosed with localized disease (stage I–III), and all underwent surgical resection as part of their treatment. Among these, two patients (cases 5 and 6) received adjuvant CAPOX: capecitabine and oxaliplatin chemotherapy and had relatively longer overall survival (12 and 29 months, respectively). One patient (case 2) initially opted for surveillance before developing metastases. A total of five patients (cases 3, 4, 7, 9, and 10) presented with metastatic disease at diagnosis, and three (case 2, 3, and 4) received immune checkpoint inhibitors (ICI), predominantly PD-1 antibodies. Among these, only one patient (case 2) achieved a durable complete response lasting 18 months. Other patients had transient responses or progressive disease despite ICI therapy. This underscores the heterogeneity and often limited efficacy of chemotherapy or ICI in SMARCA4-deficient GI malignancies.

### 3.5. Outcomes

Overall survival (OS) among reported cases ranged from as little as 2 months to a maximum of 29 months, with disease-free survival (DFS) rarely extending beyond one year except in a few resected, early-stage carcinomas (cases 2, and 5). Notably, some cases demonstrated rapid progression despite aggressive multimodal therapy, including chemotherapy and ICI, reflecting the carcinoma’s inherent resistance mechanisms. Two patients experienced disease recurrence within months of surgical resection, and one developed brain metastases (case 9) shortly after systemic therapy.

## 4. Discussion

Small intestine malignancies are exceptionally rare, constituting only 3–5% of all GI carcinomas despite the small intestine’s dominant length and surface area [12]. Among these, adenocarcinoma is the most common histologic subtype, typically presenting with localized disease and a more indolent clinical course [13]. In contrast, SMARCA4-dUTs of the small intestine are extraordinarily rare and highly aggressive, with few cases documented in the literature [12,13]. Loss of SMARCA4 expression has been increasingly recognized as a hallmark of aggressive tumor biology and is associated with poor prognosis, early metastasis, and limited response to conventional therapies [5,11].

At present, based on the limited number of reported cases, SMARCA4-deficient carcinomas of the small intestine are best considered part of a broader spectrum of SMARCA4-deficient gastrointestinal carcinomas rather than a fully distinct clinicopathologic entity. Comparison with thoracic SMARCA4-deficient carcinomas highlights both shared molecular features and important clinicopathologic differences (Table 2). Nevertheless, our review identifies recurring clinicopathologic features that may suggest a particularly high-risk subset within this spectrum.

In this review of ten published cases, several consistent patterns emerged across clinical presentation, histology, immunoprofile, and therapeutic response. Most patients presented with advanced-stage disease, demonstrated undifferentiated or rhabdoid morphology, and experienced poor outcomes despite multimodal therapy. These findings provide a focused framework for contextualizing the broader literature.

**Table 2:** Comparison of Thoracic and Small Intestinal SMARCA4-Deficient Carcinomas.

Feature	Thoracic SMARCA4-Deficient Carcinomas	Small Intestinal SMARCA4-Deficient Carcinomas
**Clinical Features**		
Primary sites	Lung, mediastinum	Mostly duodenum, other sites include jejunum, small intestine, ampulla
Demographics	Predominantly middle-aged, male predominance	Middle-aged to older adults, male predominance
Risk factors	Strong association with tobacco exposure	No consistent environmental risk factors identified
Stage at diagnosis	Frequently advanced or metastatic	Often advanced
Clinical presentation	Chest pain, cough, dyspnea, mediastinal mass	Abdominal pain, obstruction, bleeding, perforation
**Histology and Molecular Biology**		
Histologic morphology	Undifferentiated or rhabdoid carcinoma with extensive necrosis	Undifferentiated carcinoma with rhabdoid features and extensive necrosis
Cytokeratin expression	Absent or focal in most cases	Variable, some retained, others showed partial or complete loss in undifferentiated areas
Vimentin expression	Not specified	Frequently positive
SMARCA4 (BRG1)	Complete loss	Complete loss
SMARCA2	Deficient	Often lost
INI1 (SMARCB1)	Not specified	Retained
PD-L1 expression	Heterogenous levels of expression	Not enough data
**Treatment and Prognosis**		
Treatment strategies	No standard therapy	No standard therapy
Prognosis	Poor; median OS often <1 year	Poor; resected early-stage cases may show longer survival

Data summarized from published series of thoracic and gastrointestinal SMARCA4-deficient carcinomas [12,13,16,17,18,19,20,21]. Abbreviations: BRG1, Brahma-related gene 1; INI1, integrase interactor 1 (SMARCB1); OS, overall survival; PD-L1, programmed death-ligand 1.

### 4.1. Histologic and Immunophenotypic Spectrum

Most reported SMARCA4-deficient GI carcinomas display undifferentiated or rhabdoid morphologies with high-grade nuclear atypia [22,23]. In our cohort, nine of ten cases demonstrated undifferentiated or rhabdoid features, closely aligning with these established histopathologic patterns. Most carcinomas demonstrated solid sheets of poorly differentiated epithelioid cells, prominent nucleoli, irregular nuclear contours, and frequent rhabdoid features. These histologic appearances support the now well-established association between SWI/SNF complex alterations and undifferentiated or rhabdoid carcinoma phenotypes across multiple organ systems [24].

Previous studies have described a spectrum of morphologies in SWI/SNF-deficient GI carcinomas, ranging from moderate to poorly differentiated adenocarcinomas, solid or mixed histologies, to completely undifferentiated or anaplastic carcinomas with rhabdoid differentiation [25,26,27]. The cases reviewed here reflect the morphologic heterogeneity, with several carcinomas exhibiting pseudoglandular formations, scattered multinucleated giant cells, and areas of necrosis and inflammation. Notably, carcinomas involving the small intestine and ampullary region often exhibited solid, dis-cohesive growth patterns with eosinophilic or clear cytoplasm, consistent with previously reported findings [28,29,30]. Our cases showed similar growth patterns, particularly in tumors involving the jejunum and ileum, supporting this observation.

Rhabdoid morphology was a recurring feature but not universally present [31]. While rhabdoid cells are considered a hallmark of SMARCA4-deficient carcinomas, their detection may be limited by sampling issues, particularly in small biopsies. One of the primary challenges in obtaining a biopsy from a small intestine mass is its limited accessibility. The small intestine’s location within the abdominal cavity makes it difficult to access via traditional approaches. As a result, it is crucial to involve an advanced GI endoscopy team to facilitate accurate sampling and significantly enhance the diagnostic yield. Nevertheless, when observed, rhabdoid morphology serves as a useful morphologic clue prompting immunohistochemical workup for SWI/SNF complex components.

Loss of SMARCA4 protein expression by IHC was a defining feature across all cases included in this review. All ten cases in our series demonstrated complete loss of SMARCA4 by IHC, with three also showing co-loss of SMARCA2, reflecting broader SWI/SNF complex dysregulation. At present, routine testing of INI1 and SMARCA2 alongside SMARCA4 is not standardized, and available data remain insufficient to support a universal recommendation for all cases. Nevertheless, assessment of additional SWI/SNF subunits may be informative in select cases, particularly when diagnostic uncertainty exists.

No definitive morphologic standard has yet been established to distinguish SMARCA4-deficient carcinomas from other high-grade GI malignancies [13]. Indeed, some cases in this review exhibited biphasic patterns or areas of moderate differentiation, underscoring the need for ancillary testing in carcinomas with ambiguous histology. A second pathology opinion at an academic tertiary center is also essential to ensure diagnostic accuracy. The diagnostic challenge is compounded by the fact that undifferentiated carcinomas of the GI tract are rare and often poorly characterized, making awareness of this entity critical for pathologists and oncologists alike.

### 4.2. Genomic Alterations

Beyond histopathological patterns, molecular profiling provides crucial insight into the biological drivers and therapeutic vulnerabilities of these carcinomas. Genomic profiling, where available, has shown that SMARCA4-deficient carcinomas may harbor co-occurring mutations in tumor suppressor genes such as TP53. Similarly, among the small number of cases included in this review had sequencing data available, TP53 mutations were the most frequently reported co-alteration, while alterations in ERBB3 and CTNNB1 were each observed once. Other recurrent alterations included mutations in NCOR1, and DNA repair related genes such as RAD52, suggesting a complex molecular underpinning beyond SMARCA4 loss alone. These findings highlight the need for further studies to delineate cooperative genetic events that drive this aggressive tumor phenotype.

Mismatch repair testing was reported in several small intestine cases, demonstrating proficient MMR status, in line with prior literature [31]. Although rare, microsatellite instability (MSI-H) has been documented in other SMARCA4-deficient carcinomas and may have therapeutic implications. The universal MSS status in the reviewed GI carcinomas suggests that SMARCA4 loss and MSI-H are largely non-overlapping pathways in this context, though this warrants continued investigation [32].

### 4.3. Therapeutic Challenges

There is limited data on the clinical course, and treatment strategies for SMARCA4-deficient undifferentiated GI tract carcinomas [13]. Among these, small intestine carcinomas represent an underreported subset. The cases showed a pattern of frequent advanced-stage presentation, and a generally poor prognosis.

Despite the lack of standardized treatment protocols, a few patients demonstrated partial responses to ICI, including nivolumab and pembrolizumab, suggesting a potential role for immunotherapy in this setting. In small intestine cases from our review, responses to ICI varied, but those who received chemoimmunotherapy tended to show delayed progression compared to those treated with chemotherapy alone. These findings are encouraging, given the otherwise poor prognosis associated with this carcinoma subtype [17].

The variability in response to immune checkpoint inhibitors observed across the cases reflects mechanisms that have been consistently described. SMARCA4 loss has been associated with an immune-cold tumor microenvironment characterized by reduced CD8+ tumor-infiltrating lymphocytes (TILs) and higher densities of immunosuppressive cells such as regulatory T-cells and M2-polarized macrophages [33]. These likely contribute to attenuated responses to PD-1 blockade. The co-mutational profile observed in our cases may also explain for ICI heterogeneity. CTNNB1 mutations and activation of the Wnt/β-catenin pathway is among the most well-established drivers of T-cell exclusion across solid tumors. Wnt pathway activation has been shown to suppress recruitment of dendritic cells and impair priming of CD8+ T-cell responses, resulting in poor responsiveness to immune checkpoint blockade [34].

In prior studies on MSI-H colorectal cancer, pembrolizumab has demonstrated improved survival outcomes compared to chemotherapy based on Keynote-177 [35]. More recently, Checkmate-8HW demonstrated improved survival outcomes with the combination of nivolumab-ipilimumab compared to chemotherapy alone [35]. However, in our review, all small intestine cases tested for mismatch repair status were microsatellite stable. Although most of the literature focuses on thoracic SMARCA4-dUTs, emerging evidence suggests that therapeutic strategies used in lung carcinomas, such as combining anti-PD-L1 antibodies with cytotoxic chemotherapy may also be beneficial in extrapulmonary cases [32]. Interestingly, treatment responses appear to occur regardless of PD-L1 expression. In our review, PD-L1 status was negative or unknown in most cases, yet some patients experienced clinical benefit from immunotherapy. This observation reinforces the hypothesis that SMARCA4-deficient carcinomas may be immunologically responsive, even in the absence of traditional predictive biomarkers [22,23,36,37,38,39,40,41,42,43]. However, these observations are derived primarily from isolated case reports and small series, and do not establish SMARCA4 loss alone as a validated predictive biomarker for immunotherapy, particularly in microsatellite-stable disease. Accordingly, immunotherapy decisions should remain individualized and ideally discussed within a multidisciplinary framework.

**Figure 2:**
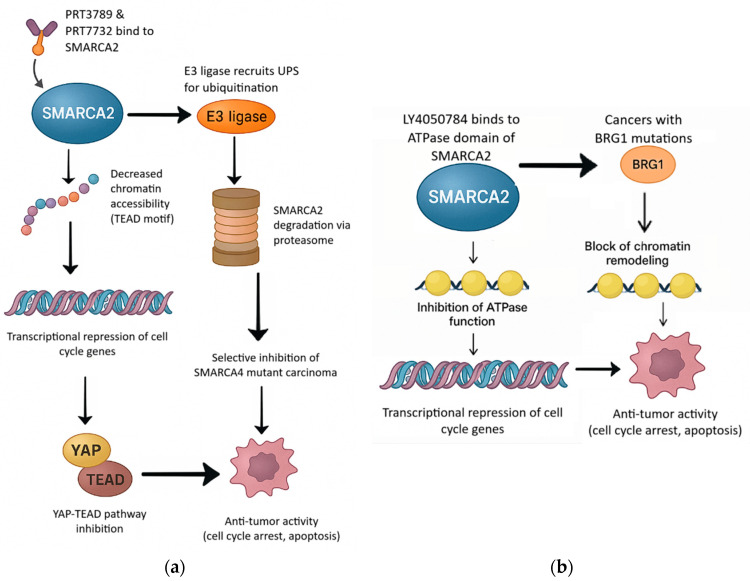
Synergistic Therapeutic Strategies Targeting SMARCA2 in SMARCA4-Deficient Cancers. Created by the authors using Google Gemini 3 and Microsoft Powerpoint (2025). (**a**) Left. PRT3789 and PRT7732: SMARCA2 PROTAC degrader: Mechanism of action of PRT3789 and PRT7732, selective SMARCA2-targeting PROTACs. These compounds bind specifically to SMARCA2 and recruit an E3 ubiquitin ligase, facilitating polyubiquitination and subsequent proteasomal degradation of SMARCA2. The degradation leads to reduced chromatin accessibility at enhancers enriched for TEAD motifs, repressing transcription of cell cycle genes via YAP-TEAD pathway inhibition. Consequently, PRT3789 and PRT7732 selectively inhibit growth and induce apoptosis in cancers harboring SMARCA4 mutations. (**b**) Right. LY4050784-SMARCA2 ATPase inhibitor: Molecular mechanism of LY4050784, a selective SMARCA2 (BRM) ATPase inhibitor. LY4050784 binds specifically to the ATPase domain of SMARCA2, blocking its function and preventing chromatin remodeling. In cancers harboring BRG1 (SMARCA4) mutations, this selective blockade results in transcriptional repression of critical cell cycle genes, leading to growth inhibition and apoptosis of BRG1-mutated cancer cells.

Prospective clinical trials are warranted to confirm these observations. One such trial is investigating the combination of tiragolumab (an anti-TIGIT monoclonal antibody) and atezolizumab in pediatric and adult patients with SMARCA4 or SMARCB1-deficient carcinomas, including those that are refractory or relapsed [44]. Other promising approaches are utiliziing SMARCA2 degraders, PRT7732, PRT3789, and LY4050784, an oral and highly potent allosteric small molecule that selectively inhibits the ATPase activity of SMARCA2 (BRM) over its closely related paralog SMARCA4 in patients with advanced or metastatic solid carcinomas harboring SMARCA4 mutations [45,46,47], Figure 2. These approaches rely on promising synthetic lethality strategies for prevalent SMARCA4 mutations and their sensitivity to SMARCA2 inhibition in lung cancer and other solid tumors [46,47].

Patients with localized disease who underwent resection followed by adjuvant chemotherapy demonstrated improved survival outcomes compared to those with unresectable or metastatic disease. Notably, a small intestine case treated with curative-intent surgery and chemotherapy remained recurrence-free beyond 12 months. However, for most patients with advanced small intestine SMARCA4-deficient carcinoma, the prognosis remained poor, with overall survival generally under 15 months and shorter progressed free interval.

Relapse or refractory disease presents a major treatment challenge. Second-line therapies varied across cases, including irinotecan, FOLFIRI, and trastuzumab-based regimens, but responses were typically limited. This highlights the urgent need for novel therapeutic strategies. Besides synthetic lethality, SMARCA2 targeting approaches, preclinical research has also suggested potential benefit from targeting epigenetic pathways, including the CDK4/6-EZH2 axis, given EZH2’s role as an antagonist of chromatin remodeling [2,48]. For example, in the Phase II pediatric MATCH trial evaluating tazemetostat, researchers focused on children and adolescents with relapsed or refractory carcinomas harboring mutations in EZH2, SMARCB1, or SMARCA4 [49]. Although EZH2 status was not routinely assessed in reviewed small intestine cases, it may represent another avenue for personalized therapy [50].

## 5. Limitations

The limitations of this review stems from the rarity of SMARCA4-deficient small intestine carcinomas and the reliance on case reports and small series, which introduces publication bias and heterogeneity in reporting. Immunohistochemical and genomic profiling were inconsistently performed across studies, limiting comparisons. Furthermore, variability in treatment approaches and follow-up periods hampers the ability to draw definitive conclusions about optimal management or prognostic markers. Importantly, overall survival could not be formally analyzed in this review because none of the included cases provided standardized or extractable OS endpoints. Although manual reference list screening was performed, the search was limited primarily to PubMed, which may have led to omission of cases indexed only in other databases.

Another limitation is the clinical and methodological heterogeneity across the case reports. Variability in diagnostic workup, pathological characterization, treatment selection, and outcome reporting introduces inconsistency that restricts the comparability of findings across cases.

## 6. Implications

SMARCA4-deficient carcinomas of the small intestine are rare and aggressive neoplasms. Recognizing this entity requires a high index of suspicion, particularly in poorly differentiated GI tract carcinomas with unusual histology, high mitotic activity, and focal rhabdoid features. Pathologists should consider SMARCA4 immunohistochemistry in cases showing loss of glandular differentiation or atypical morphology, especially when other common diagnostic entities are excluded.

Testing for SMARCA4 loss via BRG1 immunostaining is vital in carcinomas that appear undifferentiated or high-grade without clear lineage-specific features. This is particularly important when INI1 is retained, helping to distinguish SMARCA4-deficient carcinomas from other SWI/SNF-deficient mimics such as INI1-deficient rhabdoid carcinomas.

For clinicians, identifying SMARCA4 loss has both diagnostic and therapeutic implications. These carcinomas may benefit from ICI, even in the absence of PD-L1 expression or MSI-H status. However, treatment responses remain inconsistent, underscoring the need for prospective clinical trials and referral to academic institutions for multidisciplinary management. Some of the studies using synthetic lethality approaches targeting SMARCA2 in SMARC4-deficient carcinomas have been recently initiated and results are eagerly anticipated. Ultimately, early recognition and molecular workup are essential for guiding therapy and improving outcomes in this rare carcinoma subtype.

## Figures and Tables

**Table 1:** Comprehensive Summary of Cases.

Case		Age	Sex	Tumor Location	Clinical Presentation	Stage at Diagnosis	Histologic Features	IHC Staining		Molecular Testing	Treatment Administered	Tumor Response	Survival Outcome
								IHC Positive	IHC Negative				
Case 1	Akira Kambe (2024) [16]	76	M	Small intestine and adrenals	Bowel perforation	N.A.	Poorly differentiated adenocarcinoma	CK7+	CK 20- BRG-1 (SMARCA4)-	SMARCA4-	N.A.	N.A.	N.A.
Case 2	John Wang (2023) [17]	39	M	Small intestine	Intermittent abdominal pain, SBO, intussusception	T3N0M0/IIA	3.0 × 3.0 cm polypoid mass. Sheets of epithelioid and highly pleomorphic cells, many inflammatory cells, large irregular nuclei, atypical mitoses	SMA+ vimentin+ INI1+	S100- D2-40- pan-CK-CD34- CD31- ERG- DOG-1- HMB45-desmin-CD117- BRG-1 (SMARCA4)-	N.A.	Initially surveillance → later CT showed lung lesion growth, biopsy confirmed multiple mets → Pembrolizumab	Complete response in 4 months, maintained at 18 months	DFS 18 months, OS 18 months
Case 3	Shi (2024) [18]	51	M	Duodenum	Upper abdominal pain > 1 month, liver metastases, retroperitoneal LN involvement	IV	Undifferentiated tumor in cords/nests, extensive necrosis	N.A.	N.A.	SMARCA4-, PD-L1 CPS 5, MSS, TP53 and CTNNB1 mutations	Bevacizumab + Oxaliplatin + Capecitabine → PD-1 antibody + Nab-paclitaxel + Gemcitabine	Progressive disease, significant liver metastases, refused further treatment	OS 6 months
Case 4	Shi (2024) [18]	43	F	Duodenal papilla	Intermittent upper abdominal pain, supraclavicular LN enlargement	IV	Undifferentiated carcinoma	N.A.	N.A.	SMARCA4-, TP53, NCOR1, MYH9, ERBB3, RAD52, CTNNB1 mutations, TMB 10.56/Mb, PD-L1 CPS 6, MSS+	PD-1 antibody + Nab-paclitaxel + Cisplatin (6 cycles) → Intital response → Maintenance PD-1 → disease progression at 6 months → Irinotecan + Capecitabine + Bevacizumab → Supportive care	Initial response → disease progression → second line	OS 9 months
Case 5	Bin Chang (2022) [13]	54	M	Jejunum	N.A.	T3N0M0/IIA	Exophytic/endophytic 10 cm mass, diffuse sheets (85%), poor cohesive pseudoglandular (15%), epithelioid tumor cells, multinucleated giant cells, necrosis, rhabdoid cells	SMARCA2+ INI1+ ARIDIA+ P53 mutant Pan CK+ CK8+	SMARCA4-CK7-	N.A.	Surgical resection, XELOX (Capecitabine + Oxaliplatin for 8 cycles) → S1 (Tegafur + Gimeracil + Oteracil + potassium capsule for 6 cycles)	N.A.	DFS 29 months, OS 29 months
Case 6	Bin Chang (2022) [13]	34	M	Duodenal papilla	N.A.	T3N1M0/IIB	Ulcerated. Moderate adenocarcinoma (40%), undifferentiated components (60%): big nests and sheets (40%) and poor cohesive pseudoglandular (20%), epithelioid tumor cells, scattered multinucleated giant cells, necrosis.	SMARCA2(AC+) INI1+ ARIDIA+ p53 mutant Pan CK (AC+, UC+)	SMARCA4-SMARCA2 (UC-)	N.A.	Surgical resection → TACE for liver metastases, XELOX (Capecitabine + Oxaliplatin for 7 cycles) → FOLFOXIRI (Oxaliplatin + Irinotecan + Leucovorin + 5-Fluorouracil for 2 cycles)	N.A.	DFS 8 months, OS 12 months
Case 7	Bin Chang (2022) [13]	62	M	Duodenum	N.A.	T3N3M1/IV	Sheets, rhabdoid tumor cells, necrosis.	Vimentin+	SMARCA4-SMARCA2-Pan CK-	N.A.	Supportive care, no surgery or chemotherapy	N.A.	DFS 7 months, OS 11 months
Case 8	Bin Chang (2022) [13]	71	M	Duodenal ampulla	N.A.	T2N2M0/IIIB	Endophytic, nests (60%), diffused sheet (30%), poor cohesive pseudoglandular (10%), epithelioid tumor cells with focal short spindle cells, necrosis.	INI1+ ARIDIA+ p53 mutant Pan CK+ Vimentin+	SMARCA4- SMARCA2- EMA-	N.A.	Chemotherapy: GP (Gemcitabine + Cisplatin for 1 cycle)	N.A.	DFS 2 months, OS 2 months
Case 9	Bin Chang (2022) [13]	56	M	Duodenum	N.A.	T4N1M1/IV	Exophytic circular, diffused sheets (60%), nests (30%), pseudoglandular (5%), and cords (5%), broad desmoplastic stroma, mimicking desmoplastic small round cell tumor, epithelioid tumor cells (>95%), focal clear cell cytoplasm (10%), short spindle tumor cells (<5%), scattered multinucleated giant cells, necrosis, focal rhabdoid cells	INI1+ ARIDIA+ p53 mutant	SMARCA4SMARCA2-Pan CK-	N.A.	Neoadjuvant chemotherapy: TP (Paclitaxel + Cisplatin for 6 cycles) → Progressive disease, targeted therapy: Imatinib (hematochezia, anemia) → Surgery resection, postoperative MRI indicated brain tumor metastases	N.A.	OS 11 months
Case 10	Abbas Agaimy (2016) [12]	62	M	Small intestine + ampulla	N.A.	N.A.	Ulcerated transmural masses 4.5 + 5.5 cm, anaplastic large cells, rhabdoid cells	SMARCB1+ ARIDIA+ MLH1+ PMS2+ MSH2+ MSH6+ Pan CK+ Vimentin+	SMARCA4-SMARCA2- CK7- CK20- CDX2- P63-	N.A.	Resection	N.A.	OS 3 months

Abbreviations: SBO = Small Bowel Obstruction; LN = Lymph Node; N.A. = Not Available. T, N, M = Tumor size, Node involvement, Metastasis (AJCC staging system), IHC = immunohistochemistry, TACE = transarterial chemoembolization, XELOX = capecitabine and oxaliplatin, FOLFOXIRI = leucovorin, fluorouracil, oxaliplatin, and irinotecan, DFS = disease-free survival, OS = overall survival, MSS = microsatellite stable, TMB = tumor mutational burden.

## Data Availability

All data supporting the findings of this study are included in this article. Additional information may be made available upon reasonable request to the corresponding author.
